# Normalization and experimental design for ChIP-chip data

**DOI:** 10.1186/1471-2105-8-219

**Published:** 2007-06-25

**Authors:** Shouyong Peng, Artyom A Alekseyenko, Erica Larschan, Mitzi I Kuroda, Peter J Park

**Affiliations:** 1 Howard Hughes Medical Institute, Brigham and Women's Hospital, Boston, Massachusetts 02115, USA; 2 Harvard-Partners Center for Genetics and Genomics, Brigham and Women's Hospital, Boston, Massachusetts 02115, USA; 3 Department of Genetics, Harvard Medical School, Boston, Massachusetts 02115, USA; 4 Children Hospital Informatics Program, Boston, Massachusetts 02115, USA

## Abstract

**Background:**

Chromatin immunoprecipitation on tiling arrays (ChIP-chip) has been widely used to investigate the DNA binding sites for a variety of proteins on a genome-wide scale. However, several issues in the processing and analysis of ChIP-chip data have not been resolved fully, including the effect of background (mock control) subtraction and normalization within and across arrays.

**Results:**

The binding profiles of *Drosophila *male-specific lethal (MSL) complex on a tiling array provide a unique opportunity for investigating these topics, as it is known to bind on the X chromosome but not on the autosomes. These large bound and control regions on the same array allow clear evaluation of analytical methods.

We introduce a novel normalization scheme specifically designed for ChIP-chip data from dual-channel arrays and demonstrate that this step is critical for correcting systematic dye-bias that may exist in the data. Subtraction of the mock (non-specific antibody or no antibody) control data is generally needed to eliminate the bias, but appropriate normalization obviates the need for mock experiments and increases the correlation among replicates. The idea underlying the normalization can be used subsequently to estimate the background noise level in each array for normalization across arrays. We demonstrate the effectiveness of the methods with the MSL complex binding data and other publicly available data.

**Conclusion:**

Proper normalization is essential for ChIP-chip experiments. The proposed normalization technique can correct systematic errors and compensate for the lack of mock control data, thus reducing the experimental cost and producing more accurate results.

## Background

Chromatin immunoprecipitation on microarrays (ChIP-chip) is a technique that has been used primarily for investigating the binding locations of a protein on a genome-wide scale. With the availability of custom tiling arrays, this approach has yielded unprecedented resolution for these binding events. Much of the work so far has focused on binding of transcription factors [1-3], and many computational methods have been developed to identify the bound regions [4-9]. Recently this technology has been extended to the genomic mappings of other features, such as histone modifications [10], transcriptionally active regions [11], and binding sites of other protein complexes [12,13]. In the present work, we examine several issues related to the experimental design and data analysis of ChIP-chip experiments, with a focus on two-color platforms. Despite their increasing popularity [11,14], some basic analytical issues still remain unresolved.

One issue is related to the role of mock controls in the design of experiments. Mock control experiments using non-specific antibody or no antibody are often performed together with ChIP-chip experiments to control for sample handling, labeling bias, preferential amplification, and other biases that may occur in the experiment. For experiments with two-channel arrays, the immunoprecipitated sample is hybridized against the input DNA and the mock control is hybridized against the same input DNA. This design allows the same mock control to be used in multiple experiments and tends to give less noise than hybridizing directly against the mock IP, as the amount of input DNA is much larger than that of the mock IP. Without the mock control, it is possible to get false positive bound sites due to an artifact in the experimental procedure; on the other hand, the mock controls increase the experimental cost substantially and may in fact add other artifacts in some cases. The importance of this design issue was underscored in a recent paper [3]. In that work, differential enrichment of genic and intergenic regions is observed in the ChIP-chip experiments for histone occupancy in yeast. However, the mock control data also display a similar feature, and there is no substantial differential enrichment once the mock control data are used to normalize the histone occupancy data. If in general the conclusion drawn in a study depends on whether or not the mock control data are used, there is a need to perform mock control experiments in all cases, and the conclusions drawn in those experiments without mock control may be suspect. In fact, in many published works, mock control data sets are missing, and the control experiments do not appear to have been performed.

Another important and related issue is normalization of the data. There are many sources of systematic variation in microarray experiments, and normalization is a computational process for reducing the experimental artifacts, both within each array and between arrays. This issue has been studied extensively for gene expression microarray data [15,16], and the standard normalization methods for expression arrays have been extended to ChIP-chip experiments [17]. A difficulty with ChIP-chip data, however, is that the distribution of the log-ratios is asymmetric. There is only a 'bump' in the right side of the distribution, corresponding to the binding events. Standard normalization methods for expression analysis assume that there is a roughly equal number of up- and down-regulated genes or that the proportion of differentially expressed genes is small. Neither type of assumption is satisfied in general for ChIP-chip data, and standard methods do not work well. Some fixes have been proposed previously, such as mirroring the left side of the distribution to the right to estimate the background distribution [6]. However, we have noticed that the distribution of log-ratios is often not centered at zero or does not appear to have a symmetric background. A quantile normalization [16], which forces the signals in each array to follow the same distribution, has been used in some cases, but this seems to be too stringent, eliminating the variation in the degree of binding among experiments. The bias in the GC content of each probe also influences its hybridization characteristics and a normalization effect for this has been proposed recently [7].

**Figure 1:**
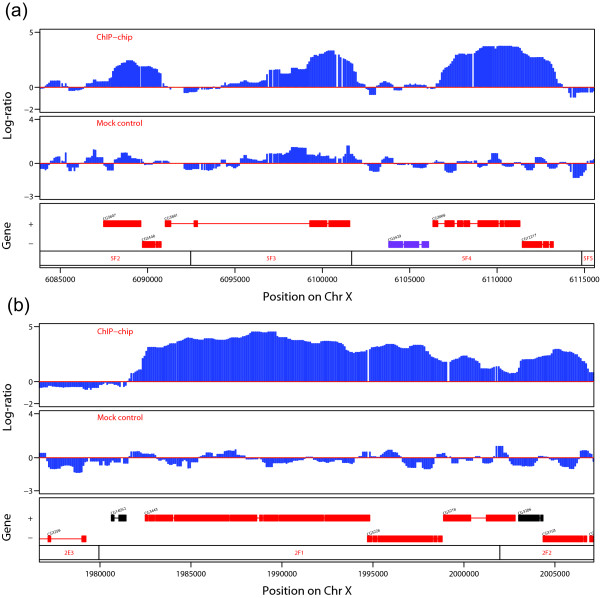
Examples of binding sites for the *Drosophila *MSL complex on the X chromosome. (a) An example of typical binding regions. (b) An example of a very large binding region (about 18 kb). Corresponding mock controls show no binding. Genes transcribed from left to right are shown on top; red genes are expressed while black ones are not.

We also note that the ChIP-chip data for chromatin-associated proteins and histone modifications present additional challenges, as they often display broad regions of enrichment. This is in contrast to the isolated and sharp peaks that are typical for the binding of transcription factors. Some examples are given in Figure 1. In Figure 1a, the log-ratios for the binding of the male-specific lethal (MSL) complex show that some regions are clearly bound, compared to the mock control. In this particular case, the bound regions appear to be between 2 kb and 7 kb; an extreme case is shown in Figure 1b, where the bound region is larger than 18 kb. The presence of these large regions mean that the typical within-array normalization schemes mentioned above are even less likely to work because of the increased asymmetry in the distribution of log-ratios. It also means that the common algorithms for finding bound regions based on Hidden Markov Models (HMMs) are likely to give incorrect results. Standard HMMs, by their construction, assume a geometric distribution of fragments, which is unlikely to hold in this data set.

In this work, we present a novel normalization method designed specifically for ChIP-chip data and show its effectiveness in correcting dye-bias and other systematic errors. We also find that proper normalization is closely related to the issue of whether mock control should be used: in the absence of proper normalization, mock control is generally necessary to eliminate the effect of systematic errors; but through appropriate normalization, the lack of mock controls may be compensated. We find that the use of the proposed normalization method alone without the use of mock control is sufficient to identify the binding events and that the correlation among biological replicates also improves as a result. Furthermore, our normalization strategy also yields insight into the question of differential enrichment of histone occupancy in genic and intergenic regions [3].

This methodological investigation is made possible by a unique data set we have generated on dosage compensation in *Drosophila *[12]. The MSL complex is known to bind specifically to the X chromosome to up-regulate the X-linked genes [12,18]. With tiling array data for the binding of the MSL complex on both the X chromosome and autosomes (2L and 4), analytical methods can be evaluated for their efficiency of identifying bound regions on the X with autosomes as control regions. This interesting feature of *Drosophila *MSL binding also offers evaluation of normalization schemes and other data analysis strategies.

## Results and discussion

### Experimental design

#### Unnormalized data require mock control experiments

**Figure 2:**
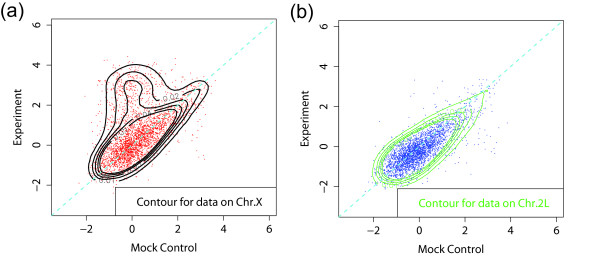
Scatter plots and density contours of log-ratios for *Drosophila *MSL complex binding. ChIP-chip experiment versus mock controls are shown for (a) chromosome X and (b) chromosome 2L, with each point corresponding to a probe. The symmetry of the scatter plot for the 2L chromosome along the diagonal *y *= *x *confirms that there is no MSL complex binding on the 2L chromosome. 3000 of the probes (about 1.5%) were randomly selected from the X and the 2L chromosomes for visualization.

To examine the role of the mock control, we first examine the MSL binding data. The data are from Nimblegen tiling arrays with 100 bp resolution (see Methods for detailed description of the arrays). In Figure 2, the scatter plots of the log-ratios for ChIP-chip versus mock control are shown, along with density contours. Figure 2a and 2b are the scatter plots for X and 2L chromosomes, respectively. The X-specificity of MSL complex binding is clearly reflected in this comparison of X and autosomes. The data points from the 2L chromosome are roughly centered on zero on both axes and are scattered around the diagonal line *y *= *x*, as expected. The deviation from this straight line is due to noise in the experiment. The symmetry of the blue region along line *y *= *x *confirms that there is no MSL complex binding on 2L chromosome and that data on 2L chromosome can be regarded as background noise. On the other hand, the data points from the X chromosome show a large number of points that have higher log-ratio in the experiment than in the mock data.

**Figure 3:**
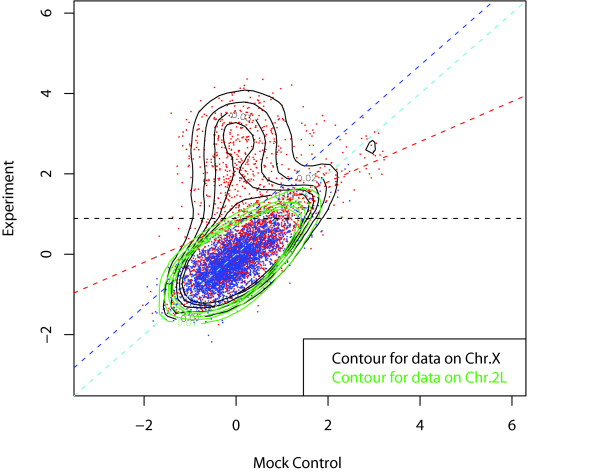
A scatter plot of ChIP-chip data versus mock control data. Data from the X (red) and the 2L (blue) chromosome were superimposed after median smoothing (window size 7) to suppress background noise. Choosing a threshold solely based on the experiment is equivalent to drawing a horizontal line, and it is clearly not optimal for separating the signal from the background. A direct subtraction of mock control from the experiment corresponds to using a line with the slope 1 as a threshold (the blue line or another parallel to it), but that also does not appear to be optimal. A better solution would be the red line, which corresponds to a subtraction with a different weight for the control.

To determine the optimal method for separating the signal from the background, we superimposed the scatter plots for X and 2L chromosome binding data in Figure 3. First, it appears that the mock data plays an important role and should not be ignored. Without them, the threshold for determining a bound region would depend only on the experiment, corresponding to a horizontal line in the figure. One may still be able to capture a significant portion of enrichment with that approach, but it would clearly result in many false positives (blue points above the horizontal line) and false negatives (red points under the horizontal line). A better method would be to utilize mock control data by subtracting the mock data from the experiment and then using a threshold on the difference. This is the typical use of the mock data in ChIP-chip studies. This direct subtraction of mock control is equivalent to drawing a line (shown as a blue dashed line) parallel to the diagonal (shown as cyan dashed line) to separate the red signal region from the blue background regions. However, the shifting of this parallel line alone, which is equivalent to changing the threshold of the log-ratio to define bound probes, cannot achieve the optimal separation (shown as red dashed line) of these two regions. Instead, it appears from the figure that further improvements can be made by a threshold that weighs the experiment and the mock data differently (this threshold corresponds to the red line). This appears to capture the shape of the background distribution from the 2L data and thus best separates the X-specific signals. Of course, the bound regions and the background regions usually cannot be separated in this way and none of the methods we develop here require such separation. But the unique characteristics of our dosage compensation system allows us to validate our methods.

#### Dye-bias and the use of mock control data

The purpose of the mock control experiment is to correct dye-bias and other systematic errors in ChIP-chip experiments. The correlation observed in Figure 2b indicates that there may indeed be some systematic bias. To examine possible dye-bias, we plot the log-ratio as a function of signal intensity. Often referred to as the 'MA plot,' we plot the log-ratios *M *= log(*R/G*) on the *y*-axis and the average intensity *A *= log(R∗G
 MathType@MTEF@5@5@+=feaafiart1ev1aaatCvAUfKttLearuWrP9MDH5MBPbIqV92AaeXatLxBI9gBaebbnrfifHhDYfgasaacH8akY=wiFfYdH8Gipec8Eeeu0xXdbba9frFj0=OqFfea0dXdd9vqai=hGuQ8kuc9pgc9s8qqaq=dirpe0xb9q8qiLsFr0=vr0=vr0dc8meaabaqaciaacaGaaeqabaqabeGadaaakeaadaGcaaqaaiabdkfasjabgEHiQiabdEeahbWcbeaaaaa@2FFA@) on the *x*-axis, where *R *and *G *are the intensities of the two-channels. This is equivalent to a scatterplot of the two channel intensities but the 45 degree rotation and rescaling makes it easier to interpret.

**Figure 4:**
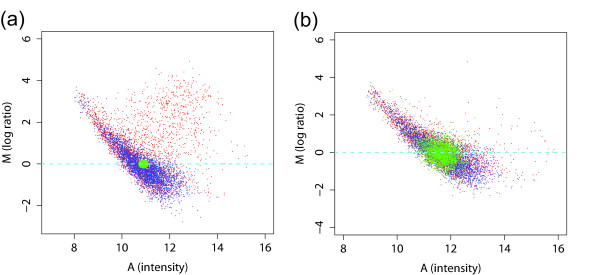
MA plots (average intensity of the signal vs log-ratio) of (a) ChIP-chip and (b) its corresponding mock control data without normalization. Both show significant dye-bias that cannot be ignored. A direct subtraction of mock control from experiment corrects the systematic errors to some extent, but it also introduces additional noise. This can be seen by how the probes marked in green in (a) are spread out in (b). Conventional within-array normalization using lowess fitting does not work well due to the presence of a large cluster of probes that show binding.

In Figure 4, the MA plots of a ChIP-chip experiment and its corresponding mock control data are shown. If there had been no intensity-dependent dye-bias, the bulk of the points should fall on the horizontal line *M *= 0. Instead, we see a striking display of intensity-dependence. Clearly, setting a threshold on unnormalized data without mock data subtraction, which would be equivalent to a horizontal line in Figure 4a, would result in a large number of false positives and negatives. Such a phenomenon has been observed in many two-color expression arrays previously and has been attributed to the differences in the size of the dye molecules and their differing efficiency, as well as nonlinearity in the scanning process [15]. The same patterns displayed in Figure 4 have been observed consistently in many tens of Nimblegen arrays. While it may not be as strong for other platforms, we have observed the same type of dye-bias in most data sets we have examined (see below).

The use of mock data is one possible solution to this problem. Because the mock data contain the same dye-bias (Figure 4b), subtraction of the mock data from the experiment data would eliminate much of the bias. However, because noise exists in both ChIP-chip and control experiments, a direct subtraction of the mock control has the disadvantage of introducing additional noise. This effect is shown in Figure 4, in which the probes in the small green region selected from the background of ChIP-chip experiment (Figure 4a) are spread out in the background of mock control experiment (Figure 4b) due to noise.

### Within-array normalization

Rather than eliminating systematic variation by subtracting the mock data, we consider the possibility of normalizing each array first. Normalization is a process for reducing the variations within and between arrays of non-biological origin [15,16]. It is an important step for any array analysis, as it has been shown that different normalization methods can lead to divergent results in expression data analysis [19]. For two-color arrays, the conventional normalization method is to fit a line through the background portion of the data in the MA plot and then to regard this line as the new horizontal line *M *= 0. Because one cannot distinguish the signal from the background, a robust line fitting method called locally weighted regression and smoothing scatterplots (lowess) [20] is often applied. This technique fits the line through the dense part of the distribution and is resistant to outliers, which are likely to be the signal. While this procedure has been shown to work well for expression arrays, it is unlikely to be effective for ChIP-chip data. The reason for this is that the signal is only positive, unlike the up- and down-regulation in expression arrays and that the amount of signal (binding) can be very large, especially for histone modifications or chromatin-associated proteins, as illustrated in Figure 1. These issues were described in the Background section. Indeed, when the lowess method is applied to our data shown in Figure 4, the fitted curve is 'pulled' by the signal (red points) and is far away from the background data (blue points). Because of the heavy bias in this data set, even a more robust version of lowess, such as one based on interquartile range of residuals, is unlikely to work near the high-intensity spots. Other line fitting methods we have tried also gave the same unsatisfactory results. While it is not difficult to see where the line should be from visual inspection, the problem of fitting a line through the background when the background is mixed with the signal is not trivial.

**Figure 5:**
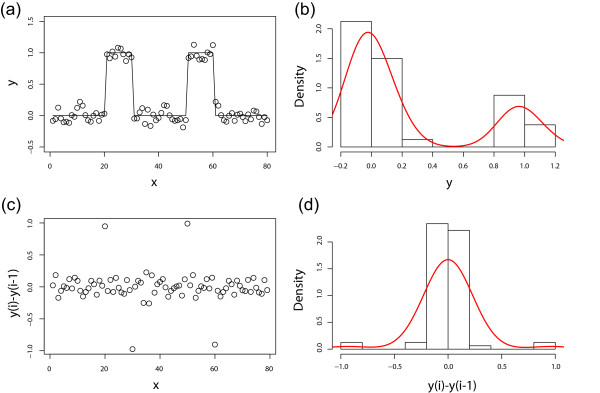
Illustration of the key idea. When the log-ratio profile shows binding for a large fraction of probes (a), the distribution of log-ratios has a heavy tail on the right side (b). But the profile of the differences between neighboring probes (c) are not sensitive to the amount of binding. The few points that correspond to the jumps in (a) are large but are now distributed evenly between positive and negative values, and the distribution of the differences is symmetric (d).

We propose a novel method to solve this issue. The main idea is that we perform the line fitting not on the original data but on the first order *differences *of the probe values along their chromosomal location. This is a discrete version of taking a derivative. This idea is illustrated in Figure 5 with a simulated profile. When there is a binding in the profile (Figure 5a), the distribution of log-ratios has a 'bump' on the right side of the distribution (Figure 5b). But when we look at the differences of neighboring probes, the beginning and the end of the bound region give a positive and a negative value, respectively, but the middle of the bound region is indistinguishable from unbound regions (Figure 5c). That is, the distribution of the log-ratios is *symmetrized*.

**Figure 6:**
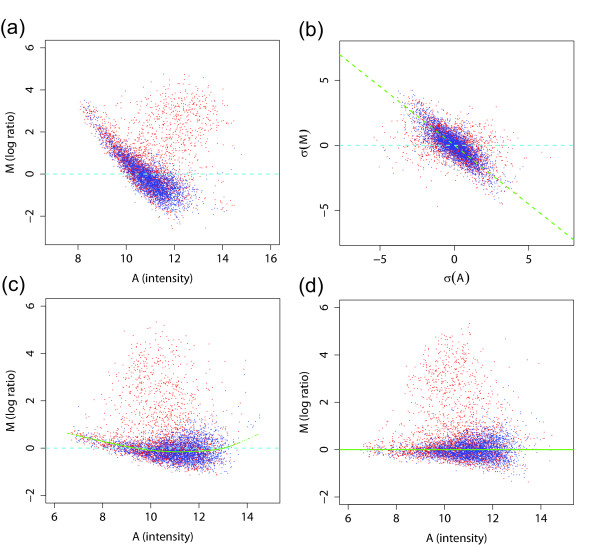
The proposed normalization procedure: (a) The MA plot before normalization shows a need for rotation to correct dye-bias. (b) To determine the correct angle of rotation, the *σ*(*M*) vs *σ*(*A*) plot of the differences between probes is generated (the differences were taken between probes that are 800 bp along the chromosome; see Figure 8). This circumvents the effect of binding signal in determining the rotating angle for original MA plot in (a). (c) The MA plot after rotation by the angle determined in (b). The green line is the lowess fitting line after rotation. (d) The MA plot after lowess normalization. 3000 sample points from each of X and 2L chromosomes are shown.

Our novel normalization procedure based on the above idea is illustrated in Figure 6. First, when the bias is strong, as is the case here, we rotate the data in the MA plot rather than fitting a lowess curve first. Based on the scatterplot, measuring the deviation perpendicular to the background appears reasonable, and this also ensures that the signals do not dominate the background at all different intensities. To select the rotating angle from the MA plot, we transform the data to the differences between neighboring probes for both *M *and *A*, denoted by *σ*(*M*) and *σ*(*A*) (Figure 6b). This *σ*(*M*) vs *σ*(*A*) plot shows the same dye-bias trend as the MA plot, but signal portion is scattered in a symmetric manner as to not influence the determination of the rotating angle, regardless of the amount of the signal. After rotation (Figure 6c), we can perform the standard lowess-type normalization for any remaining nonlinear artifacts. In order to further minimize the effect of signal in the line fitting, outlier log-ratios can be given smaller weights. In our example, for instance, those log-ratios (M) more than two standard deviations away from their median were given zero weights in the lowess fitting. We also employed an iterative procedure in which the outliers were redefined after each step and the curves were refit, but it gave similar results. Figure 6d shows the final MA plot after normalization steps.

**Figure 7:**
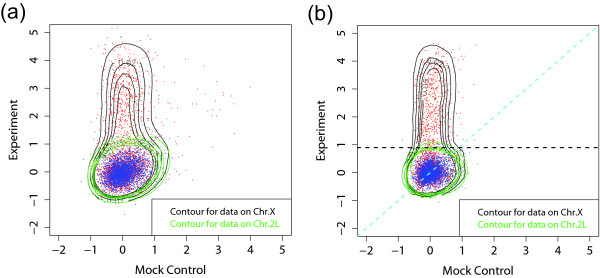
The Scatter plot of ChIP-chip versus mock control after rotation and lowess normalization (cf. Figure 3), (a) without and (b) with running median smoothing. The optimal line for separating the X-specific signals from the 2L signals is now a horizontal line. This suggests that mock control data correction may be neglected when a proper normalization is carried out.

After this normalization, the scatterplot between experiment and mock control changes dramatically, as shown in Figure 7a (cf. Figure 3). With the reduction of the systematic variations, the log-ratios have a small deviation, and a horizontal line is now sufficient to separate the signal from the background. This suggests that correcting the ChIP-chip data using mock control may be neglected after a proper normalization.

The combinatorial method of rotation and lowess normalization with no use of mock control data in fact results in improved correlations among biological replicates compared to the subtraction of the mock data, both at the probe level and at the gene level. For the two ChIP-chip replicates for two cell types (*Drosophila *embryos and Clone 8 cells), the correlations at the probe level increases from 73% and 78% to 82% and 79%, respectively. For the agreement for enrichment at the gene level, the matches increase from 89% and 86% to 96% and 95%, respectively. This may be partly due to the reduction in the noise level as a result of avoiding the subtraction of log-ratios. A further improvement in the identification of the signal can be made by simple smoothing of the profiles (see Figure 1) along their chromosomal location, for both ChIP-chip and the mock control. Regardless of the algorithm used for finding bound regions, the smoothing step suppresses the effect of outlier probes and results in improved performance. The amount of smoothing applied depends on the type of binding sites and the spacing of the arrays. In our case, a running median smoothing along each probe with a window size of 7 probes gave good results. In contrast to Figure 7a, there are fewer stray probes in Figure 7b and both the signal and background parts appear to be 'tighter' with smaller standard deviations.

### Measurement of noise levels and normalization across arrays

In the previous section, we used the differences of log-ratios from neighboring probes as a means to symmetrize the distribution and perform curve fitting. But the same idea can be used to estimate the level of array-specific noise, which can be used to normalize between arrays. As shown in Figure 5, the differences of the consecutive probes is not affected by the amount of binding, other than few outlier points corresponding to the ends of the bound regions. Thus, a measure of deviation for the distribution of differences (Figure 5d) would serve as a reasonable measure of noise. The median absolute deviation can be used to obtain a robust measure of deviation (see Methods).

**Figure 8:**
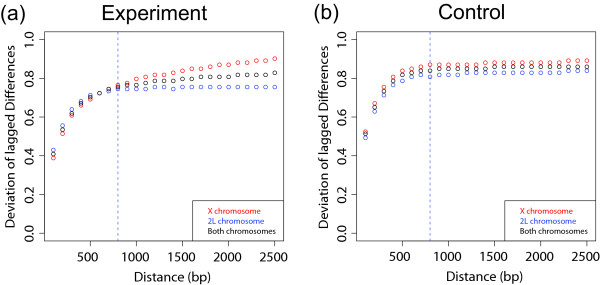
Measurement of within-array noise using lagged differences. Generalizing the concept of differences on probe values along the chromosomal location, median absolute deviation is measured for probes that are apart by the distance shown on the *x*-axis. In both experiment and control, after about 800 bp, there is no spatial correlation, and this deviation can be used as a noise level for standardizing across arrays.

More generally, we can study the spatial correlation between neighbors *i *and *i *+ *j*, for some *j *≥ 1. Because sonicated fragments are being hybridized, there is still correlation between consecutive probes (*j *= 1). As the probes farther away are compared, this correlation weakens and, conversely, the estimate of the background noise increases. In our data, we have found that after *j *≈ 8, the correlation is significantly diminished and the noise estimate becomes relatively stable. This is illustrated in Figure 8 for both the experiment and the mock control. With 100 bp resolution on our arrays, *j *= 8 corresponds to a distance of 800 bp, which is roughly the size of the DNA fragments.

To see how the amount of binding affects this estimate, the noise estimates were obtained separately for chromosomes X and 2L as well as the combined data (Figure 8a). For chromosome 2L, there is a clear stabilization of the noise estimate; for chromosome X, it continues to increase due to the long range correlations but the increase is very slight. For the mock control, there is almost no distinction between the X and the 2L chromosomes and they reach a plateau around *j *= 8. The proximity of the curves near *j *= 8 for X and 2L indicate that the proposed estimate of noise is resistant to the different amount of binding. This estimate can therefore be used to rescale the log-ratios explicitly or as a basis for determining a significance threshold on each array.

### Validations

To determine the effectiveness of the proposed normalization, we examine how the analysis results change between this and the standard mock subtraction normalization. This is studied in the context of two data sets.

#### Identification of binding sites by the MSL complex

**Figure 9:**
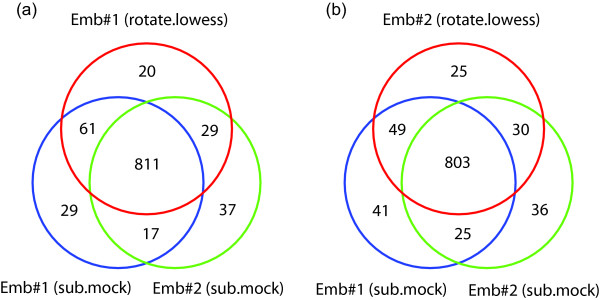
Comparison of binding sites identified by direct subtraction of mock control and the proposed normalization without mock control. The two biological replicates for MSL binding in embryos (blue and green) show a large overlap (85%) in the identified sites. The new normalization method involving rotation and lowess-curve fitting (red) was applied to each replicate. In both cases, the new method identifies nearly all of the common sites (98% and 97%) found by direct subtraction and many of the sites found by only one. Also, the new method for sample 1 gives a greater percentage of overlap with mock-subtracted sample 2 than mock-subtracted sample 1 does. Similarly, the new method for sample 2 gives a greater percentage of overlap with mock-subtracted sample 1 than mock-subtracted sample 2 does.

We had previously used mock subtraction for our analysis and identified several hundred binding sites that show up repeatedly in biological replicates [12]. In the current analysis, we seek to determine the overlap between the sites identified from the old and the new analyses, using *Drosophila *embryo data as an example. In Figure 9, we display Venn diagrams that show the overlap of the clusters obtained between the two replicates using the mock subtraction analysis and each of replicates using the new analysis. First, we see that the overlap between the two replicate was already high in the previous analysis (blue and green), with the size of the intersection over the size of the union being (811 + 17)/(811 + 17 + 61 + 29 + 29 + 37) = 85%. With the new analysis (red), most of the overlapping binding sites are reproduced (811/(811 + 17) = 98% and (803/803 + 25) = 97%). To determine whether the new method can be a substitute for the old one, we compare the result of (i) Embryo sample 1 (proposed method) vs Embryo sample 2 (mock subtraction) with that of (ii) Embryo sample 1 (mock subtraction) vs Embryo sample 2 (mock subtraction). As shown in Figure 9a, the result is in fact slightly better for case (i). There are more overlapping binding sites in case (i) than in case (ii), 811 + 29 vs 811 + 17, and fewer non-overlapping binding sites in case (i) than in case (ii), 61 + 20 vs 61 + 29. That is, the bound genes obtained by the normalization method without the mock data is at least as accurate as those obtained with mock subtraction. A similar result is obtained for the other Embryo sample (Figure 9b). To make it fair, roughly the same number of clusters had to be chosen in each case in this comparison. Hence, the number of probes called significant was equalized by taking the same top 13% of the log-ratios from the X chromosomes, which corresponded to the number of sites found in the original work [12]. The clusters were then formed based on these probes.

#### Histone occupancy and modification

**Figure 10:**
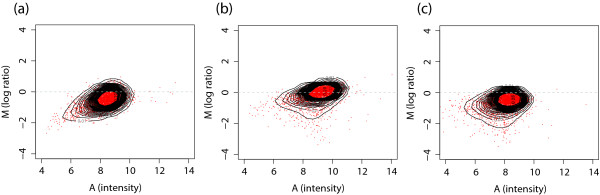
The comparison of MA plots for (a) mock control (with no antibody), (b) histone occupancy (with anti-histone H3), and (c) histone modification (with anti-histone H4) ChIP-chip experiments. The mock control experiment shows a different dye-bias from other ChIP-chip experiments. This suggests that the dye-bias needs to be corrected before using this mock control to normalize ChIP-chip experiments through subtraction.

In Pokholock et al [3], the authors generated genome-wide maps of nucleosome acetylation and methylation patterns in yeast. They examined the relationship between the modification status of various histones and transcriptional activity, and they associated specific events with different parts of actively transcribed genes. Agilent arrays were used, with 44K 60-mer probes covering 12 Mb (85%) of the yeast genome except the highly repetitive regions and an average probe density of 266 bp. Importantly, the authors note their surprise in finding that while differential enrichment of intergenic and genic regions was observed in histone occupancy, the same was also observed in control experiments without antibody or antibody recognizing a nonhistone protein, possibly due to the different relative levels of intergenic and genic DNA recovered during extraction procedures. When these controls experiments were used to normalize the histone H3 data, they found that the difference between the relative levels of intergenic vs genic DNA was not substantial. The authors show that the use of a different control explains the discrepancy between their conclusions and that of an earlier paper [21], in which genomic DNA was used. To investigate whether the above observation is affected by the statistical technique used for normalization, we applied the proposed normalization method to the same data set. In Figure 10, MA plots are shown for mock control (with no antibody), histone occupancy with anti-H3 antibody, and histone occupancy with anti-H4 antibody. Although not as strong as in the *Drosophila *data set above, there is a substantial dye-bias that requires correction, especially for the no-antibody case. It is clear that a direct subtraction of the mock data to correct dye-bias in ChIP-chip data can introduce additional noise in the analysis. There is no obvious cluster of points with positive log-ratios corresponding to the binding this time, but the signal is still present.

**Figure 11:**
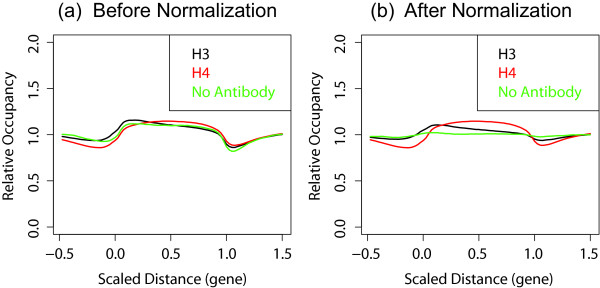
Composite profiles over 5527 genes for histone occupancy (with anti-histone H3 or H4) and mock control (with no antibody) in yeast [3], (a) before and (b) after normalization. The boundaries of ORFs were defined by transcription start and stop sites and were scaled to [0, 1]. The upstream and downstream regions are plotted with the same scale with the ORF. Before normalization, the mock control appears to show the same differential enrichment between genic and intergenic regions as the histone occupancy, suggesting that the differential enrichment may be an artifact. However, after normalization, the mock control no longer shows significant differential enrichment while H3 and H4 profiles still do.

The binding pattern across all rescaled genes is shown in Figure 11. Without any normalization, both histone occupancy and mock control data show differential enrichment of genic regions over intergenic regions, as described in the original manuscript [3]. However, when the data are normalized with rotation and lowess-fitting, the no-antibody case no longer shows differential enrichment while the H3 and H4 cases still show it. This lends a strong support to the conclusion that the differential enrichment exists and that the problem of a control experiment having the same enrichment pattern may have been an artifact.

## Conclusion

The X-specificity of *Drosophila *MSL complex binding provides an excellent opportunity for the development of methods for identifying bound regions in ChIP-chip data. We have found that there is significant bias due to the differences in the dye, as seen on the average intensity vs log-ratio plot, and that this must be corrected to obtain accurate estimates of binding sites. One way to fix this problem is via direct subtraction of the mock control, but it appears that the lack of mock control may be compensated through proper normalization steps. We developed a normalization procedure for ChIP-chip experiments based on the differences of the neighboring probes along the chromosome and found that it improves both the correlation of log-ratios at probe level and the overlap at gene level among replicates. Conventional normalization methods may work for ChIP-chip experiments with transcription factors because the proportion of the bound probes is generally small. But with histone modifications or binding of chromatin-associated proteins, that proportion may be much greater and the standard methods do not work well. We also used the same idea to measure array-specific noise for normalization across arrays.

We have examined the Nimblegen and Agilent platforms in detail here, but other studies using these and other two-dye platforms are likely to have experimental artifacts. It is thus important that the data are processed properly to obtain accurate description of the binding sites.

## Methods

### ChIP-chip data sets

Data from two *Drosophila *male cell types (Clone 8 and SL2) and late-stage embryos are available for the experiments with the MSL complex. Because the MSL complex appears to bind to some regions on autosomes in SL2 cells, possibly due to genome rearrangement, we used the embryo data for developing and testing data analysis methods. The custom NimbleGen arrays for these experiments contained 388,000 probes each and were designed based on FlyBase 3.2. The X and the 2L chromosomes were tiled with 50-mer probes every 100 bp except for the repetitive regions. For the ChIP-chip experiments, tandem affinity purification (TAP) tag was added to the C-terminus of the MSL3 protein, which is a component in the MSL complex. The addition of the TAP-tag does not affect the function of MSL complex and chromatin immunoprecipitation was achieved by an antibody specifically recognizing the TAP tag. Mock control experiments were done following the same protocol as the ChIP-chip experiments but in the absence of the TAP-tagged MSL complex (detailed in [12]). All data are available from the authors' web site. The array designs and the ChIP-chip data for histone modification and occupancy in yeast [3] were obtained from the ArrayExpress database under the accession number E-WMIT-3.

### Identification of binding sites

To measure the noise level in each array, we employed the median absolute deviation to the lagged differences. This is defined as σ* (*j*) = *s *× median|*d*_*ij *_- median(*d*_*ij*_)|, where *d*_*ij *_= *x*_*i *+ *j *_- *x*_*i *_and *x*_*i *_is the log-ratio of the *i*th probe. The scaling factor *s *≈ 1.4826/2
 MathType@MTEF@5@5@+=feaafiart1ev1aaatCvAUfKttLearuWrP9MDH5MBPbIqV92AaeXatLxBI9gBaebbnrfifHhDYfgasaacH8akY=wiFfYdH8Gipec8Eeeu0xXdbba9frFj0=OqFfea0dXdd9vqai=hGuQ8kuc9pgc9s8qqaq=dirpe0xb9q8qiLsFr0=vr0=vr0dc8meaabaqaciaacaGaaeqabaqabeGadaaakeaadaGcaaqaaiabikdaYaWcbeaaaaa@2DB9@ is introduced so that the σ* becomes the standard deviation *σ *when the underlying distribution is normal. There are two parameters to consider in determining whether a region is bound: the threshold for significant log-ratio value and the minimum number of probes needed to define a cluster of probes. We set the threshold for log-ratios to 2σ*(*j *= 8) to define "enriched" signal probes (note that this definition is different from the one in Ref [12] due to the lag). We also set the minimum number of clusters to be 8, based on the correlation length of 800 bp obtained from Figure 8. These values give a high enrichment ratio between the number of the sites on the X and the 2L chromosomes and the false discovery rates based on random permutation are < .05.

## Authors' contributions

SP performed the analysis, AAA and EL performed biological experiments and provided biological insights, MIK and PJP supervised the project, and SP and PJP wrote the paper. All authors read and approved the final manuscript.
